# How to Treat the Ear

**Published:** 1891-01

**Authors:** 


### HOW TO TREAT THE EAR.

It is an old saying that one should never put any thing smaller than his elbow into his ear. Let us add, do not put any thing cold into the ear; even cold water should be avoided, especially if there is any affection of the hearing. Do not put cotton in the ears if there is any discharge of pus from them. Use warm water as frequently as may be necessary to keep them clean, but do not force the foul matter back into the delicate machinery. If any small hard substance falls into the ear, do not attempt to "dig it out." If not readily removable, allow it to remain in quiet, and get a surgeon to see to it; it is not likely to do any serious harm unless tampered with. Deafness may sometimes be caused by an excess of ear-wax, which has become hardened and obstructs the action of the membrane. Either have a careful hand apply warm water through a proper syringe, or a piece of cotton wadding made wet with essence of peppermint may be introduced, which will dissolve and absorb the hardened wax in a few hours.

